# Minimally invasive reconstruction of lateral tibial plateau fractures using the jail technique: a biomechanical study

**DOI:** 10.1186/1471-2474-14-120

**Published:** 2013-04-04

**Authors:** Andre Weimann, Thomas Heinkele, Mirco Herbort, Benedikt Schliemann, Wolf Petersen, Michael J Raschke

**Affiliations:** 1 Department of Trauma-, Hand- and Reconstructive Surgery, University Hospital Münster, Münster, Germany; 2 Department of Traumasurgery, Martin-Luther-Hospital, Berlin, Germany

**Keywords:** Tibial plateau fractures, Jail technique, Osteosynthesis, Displacement, Load, Stiffness, Failure

## Abstract

**Background:**

This study described a novel, minimally invasive reconstruction technique of lateral tibial plateau fractures using a three-screw jail technique and compared it to a conventional two-screw osteosynthesis technique. The benefit of an additional screw implanted in the proximal tibia from the anterior at an angle of 90° below the conventional two-screw reconstruction after lateral tibial plateau fracture was evaluated. This new method was called the jail technique.

**Methods:**

The two reconstruction techniques were tested using a porcine model (n = 40). Fracture was simulated using a defined osteotomy of the lateral tibial plateau. Load-to-failure and multiple cyclic loading tests were conducted using a material testing machine. Twenty tibias were used for each reconstruction technique, ten of which were loaded in a load-to-failure protocol and ten cyclically loaded (5000 times) between 200 and 1000 N using a ramp protocol. Displacement, stiffness and yield load were determined from the resulting load displacement curve. Failure was macroscopically documented.

**Results:**

In the load-to-failure testing, the jail technique showed a significantly higher mean maximum load (2275.9 N) in comparison to the conventional reconstruction (1796.5 N, p < 0.001). The trend for better outcomes for the novel technique in terms of stiffness and yield load did not reach statistical significance (p > 0.05). In cyclic testing, the jail technique also showed better trends in displacement that were not statistically significant. Failure modes showed a tendency of screws cutting through the bone (cut-out) in the conventional reconstruction. No cut-out but a bending of the lag screws at the site of the additional third screw was observed in the jail technique.

**Conclusions:**

The results of this study indicate that the jail and the conventional technique have seemingly similar biomechanical properties. This suggests that the jail technique may be a feasible alternative to conventional screw osteosynthesis in the minimally invasive reconstruction of lateral tibial plateau fractures. A potential advantage of the jail technique is the prevention of screw cut-outs through the cancellous bone.

## Background

Fractures of the tibial plateau are severe injuries, accounting for 5-8% of all fractures of the lower leg. The most frequent reasons for these injuries are falls, traffic accidents and sports trauma. In recent years, the incidences of these fractures have risen due to increase in motorization and alternative sport activities and an increasingly aging population [1].

Tibial plateau fractures may occur as an effect of axial load transmission into the condyle of the tibia, with the lateral part of the tibial plateau more often affected. According to Holz et al., the exposed position of the lateral tibial plateau is mainly the reason for the higher incidence of fractures in this part of the bone [2].

Burst fractures of the tibial plateau are characterized by problematic healing due to high complication rates, instability of fixation and complex fracture patterns [3]. To address these challenges in fracture management, the use of minimally invasive fixation techniques has become popular in recent years [3,4]. However, comparative biomechanical data on these techniques are scarce.

In literature, many studies have compared the clinical outcomes after minimally invasive treatment of burst tibial fractures [5,6]. Good clinical results have been reported especially following arthroscopic-assisted minimally invasive reconstruction. In these reports, the importance of exact reposition of the joint surface and the joint congruence is emphasized. Even in burst fractures with <2 mm gap, a minimally invasive operative procedure is recommended [7,8].

**Figure 1:**
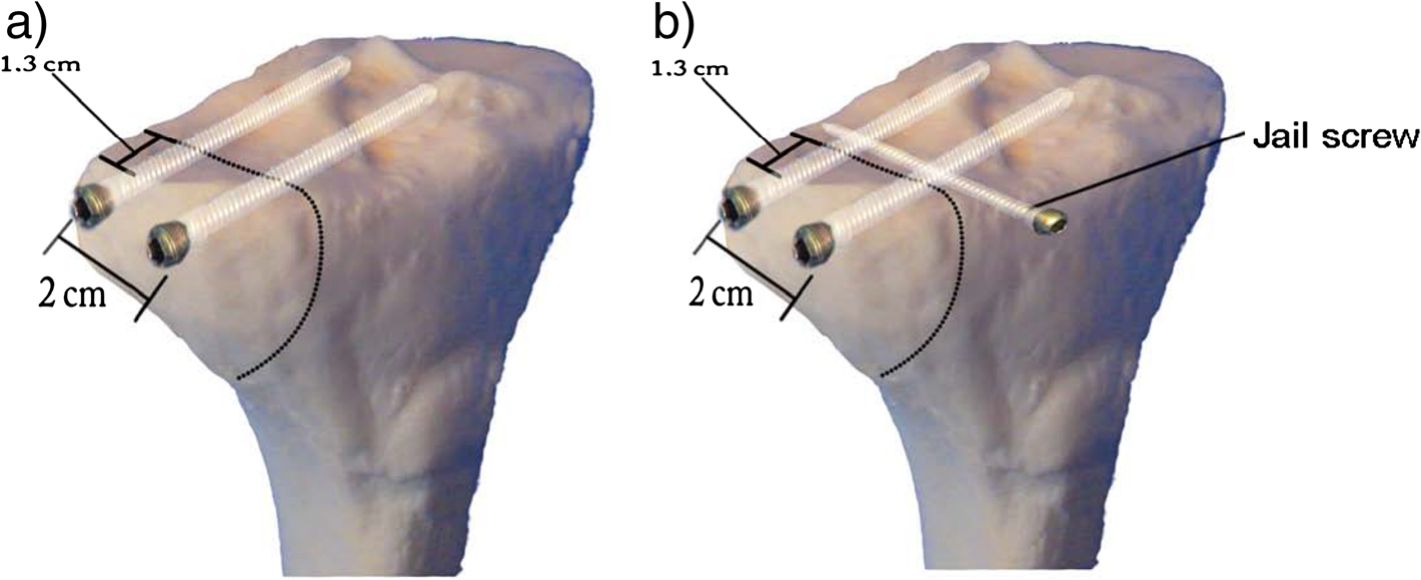
**The two different reconstruction techniques used in this study.** The schematic drawing shows the two techniques used in this study; **a**) conventional reconstruction using a lateral two-screw osteosynthesis; **b**) jail technique.

This biomechanical study compares two minimally invasive techniques for the stabilization of lateral tibial plateau fractures, with focus on fractures classified as AO 41 B1. Burri et al. suggested a two-screw osteosynthesis technique for these kinds of fractures [9]. In this paper, we describe the so-called “jail” technique which combines the osteosynthesis method with an additional screw implanted in the proximal tibia from the anterior at an angle of 90° below the two lateral screws as a counter bearing (Figure 1). The arrangement of screws resembles a prison grating, from where the term “jail” technique was derived. Biomechanical data for this novel technique are not available in literature. Both conventional and jail techniques may be performed in an arthroscopic-assisted manner.

This comparative study aims to test the hypothesis that the additional third screw inserted anteriorly can better restore structural properties compared to the well-known two-screw osteosynthesis.

## Methods

### Specimens

**Figure 2:**
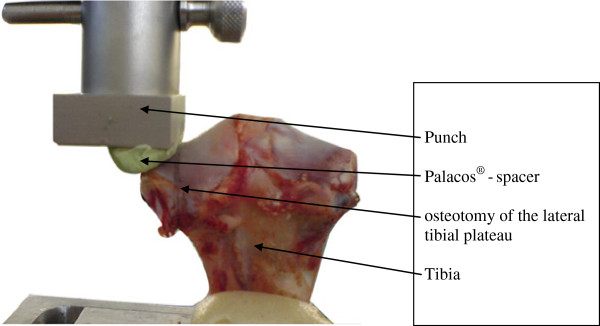
**The experimental setup.** The figure shows the porcine model with osteotomy of the lateral tibial plateau set up in the material testing machine Palacos® - spacer.

Forty fresh skeletally mature porcine tibias were obtained from a local butcher and frozen at −20°C. The porcine model was selected because of the similarities of structural properties between the human and the porcine knees [10]. The specimens were thawed for 12 hours at room temperature and all muscles and soft tissue were removed before testing. After cleaning and degreasing the tibial diaphysis, the lower part was embedded in a metal container using a two-component polyurethane foam with the tibial plateau in an upright position. A standardized osteotomy was performed to simulate fracture of the lateral tibial plateau. The holes for screw osteosynthesis as well as the additional jail screw were drilled before osteotomy. This was to ensure that an exact reposition of the joint surface and the joint congruence after osteotomy was possible. The drill holes had a diameter of 3 mm and were localised 7 mm under the joint line with a 20-mm distance. The osteotomy was performed with an oscillating saw at a distance of 13 mm from the lateral tibial plateau (Figure 2).

### Reconstruction techniques

Following osteotomy, two different screw fixation techniques for lateral tibial plateau fractures were biomechanically evaluated: the conventional two-screw osteosynthesis (Figure 1a) as described by Büri et al. in 1979 [9] and the jail technique which introduced another screw transverse to osteosynthesis screws as shown in Figure 1b. In the latter, the third screw was placed in an angle of 90° below the two conventional screws in the intact part of the bone next to the fracture site. To ensure that the supporting lower screw is in direct contact with the 2 upper lag screws, a 1-mm K-wire was placed under the two screws and used as a screw guide wire.

For each single specimen, an individual spacer was created using a defined amount of 10 ml bone cement. This spacer was placed right between the punch of the material testing machine and the lateral tibial joint to make sure that an optimal contact surface was achieved. The spacer was made of Palacos® (Heraeus Medical GmbH, Wehrheim, Germany) a two-component bone cement (Figure 2). The whole construct (orientation of the punch, tibial slope, distal tibia) was adjusted in an uniaxial direction to ensure that the conditions at the repair site were repeatable in every individual test setup. This model was chosen after preliminary testing of a control group adjusting the spacer-joint surface interface in an original lateral tibial plateau.

Each individual spacer underwent an axial loading up to 3000 N for 10 minutes prior to testing to confirm that there is no relevant deformation of the spacer in the test setup.

### Testing protocol

The two tibial fracture reconstructions were subjected to two different loading protocols. In the first protocol, both reconstructions (n = 10 per group) underwent a load-to-failure testing protocol using a material testing machine (Zwick/Roell® Z005, Zwick Gmbh & Co. KG, Ulm, Germany). During load-to-failure testing, an axial load was applied with a loading rate of 200 mm/min on the reconstructed fragment of the lateral tibia simulating a worst case scenario (Figure 2). As a second test, a cyclic loading ramp protocol (n = 10 per reconstruction group) was introduced using an INSTRON® 8874 servo-hydraulic material testing machine (Instron® Deutschland GmbH, Pfungstadt, Germany). A preload of 30 N was first applied to all specimens. All reconstructions were cyclically loaded in 5 steps: 30-200 N, 30-400 N, 30-600 N, 30-800 N and 30-1000 N, 1000 cycles in each step. Cyclic loading was performed at a displacement rate of 200 mm min^-1^ and a loading frequency of 80 cycles min^-1^. This loading protocol is within the general range reported in previously published studies involving cyclic loading, and represents a relatively modest load level imitating an aggressive rehabilitation protocol [11]. The loading frequency was similar to that of other studies and appears to be within a physiological range of loading [10].

Reconstructions that survived the cyclic loading protocol were finally tested until failure in the same test setup. A total of 40 porcine tibias were used, with each tibia used only for one test setup. All tests were performed at room temperature, and the tibias were kept moist during preparation, mounting, and testing to prevent desiccation with saline.

During the testing process all data was recorded continuously using a computer data recording system. After testing maximum load, yield load, stiffness and displacement were analyzed. Additionally a load displacement curve was recorded. Maximum load was defined to be the highest measurable value of the load displacement curve. Failure (yield load) was defined as the point in the curve where the reconstruction failed and started to undergo plastic deformation.

Stiffness was defined as the slope of the linear region of the load displacement curve.

**Figure 3:**
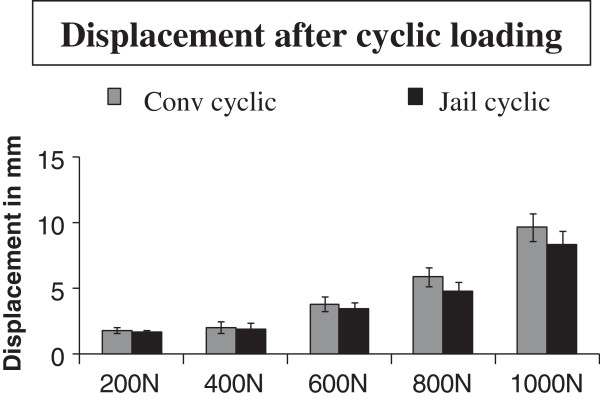
**The load displacement curve.** This figure shows the load displacement from the recorded data during the testing protocols. The ordinate (y-axis) is the measured displacement recorded in mm. The abscissa (x-axis) shows the expressed load as force in newtons (N).

Displacement was analyzed after the last cycle of each loading step in the ramp protocol (Figure 3) and after loading to failure at the end of every test setup.

Failure mode was monitored by visual analysis, photo and video documentation.

### Statistical analysis

Statistical analysis was performed using the SPSS 14.0 software (SPSS Inc., Chicago, Illinois, USA). The *T*-test was used to compare differences between reconstruction groups and the Levene´s- test to assess the equality of variances between samples. Significance was set at p <0.05.

## Results

### Single cycle loading protocol

In the single cycle protocol, all reconstructions in both groups tested were loaded until failure. Reconstructions using the conventional osteosynthesis showed a significantly lower maximal load (1769.46 ± 107.47 N) in comparison to the jail technique (2275.87 ± 253.33 N) (p < 0.001).

There was a tendency of better outcomes using the jail technique for stiffness (conventional osteosynthesis = 441.61 ± 98.211 N mm^-1^; jail technique = 491.81 ± 98.54 N mm^-1^) and yield load values (conventional osteosynthesis 1068.73 ± 110.29 N; jail technique 1154.7 ± 199.92 N) but these results were not statistically significant (p = 0.228, 0.150, respectively).

On the other hand, displacement measurements tended to be lower using conventional osteosynthesis compared to the jail technique but this result did not reach statistical significance (Table 1).

**Table 1:** Results of the single cycle load-to-failure protocol

**Variable**	**Mean ± SD (N)**		**p-value**
	**Conventional osteosynthesis**	**Jail technique**	
	**n = 10**	**n = 10**	
Maximum load	1796.46 ± 107.47	2275.87 ± 253.33	<0.001*
Displacement	4.49 ± 0.71	4.94 ± 1.65	0.903
Stiffness	441.61 ± 98.21	491.81 ± 98.54	0.228
Yield load	1068.73 ± 110.29	1154.69 ± 199.92	0.150

* significant at p < 0.05.

### Cyclic loading protocol

In the second testing protocol, all reconstructions loaded cyclically survived the ramp protocol and were afterwards loaded to failure in the same test setup.

During the cyclic testing, the displacement of the lateral tibial fragment was analyzed. In all 5 steps, reconstructions using the jail technique showed lower displacements in comparison to the conventional osteosynthesis (Table 2). The mean displacement after 5000 cycles was 9.54 ± 1.84 mm for the conventional osteosynthesis and 8.53 ± 1.68 mm for the jail technique. The results, however, did not reach statistical significance.

**Table 2:** Results of the cyclic loading protocol

**Variable**	**Mean ± SD (mm)**		**p-value**
	**Conventional osteosynthesis**	**Jail technique**	
	**n = 10**	**n = 10**	
Displacement 200 N	1.38 ± 0.55	1.21 ± 0.40	0.596
Displacement 400 N	2.22 ± 0.56	2.02 ± 0.49	0.693
Displacement 600 N	3.36 ± 0.63	3.09 ± 0.70	0.686
Displacement 800 N	5.63 ± 1.34	5.12 ± 0.88	0.745
Displacement 1000 N	9.54 ± 1.84	8.53 ± 1.68	0.530

Looking at the loading to failure after cyclic loading to evaluate the residual stability of the reconstructions, the result showed again a trend for better outcomes in terms of maximal load, yield load, stiffness and displacement for jail technique (Table 3). The differences, however, were not statistically significant.

**Table 3:** Results of the load-to-failure protocol on specimens which survived the cyclic loading protocol

**Variable**	**Mean ± SD (N)**		**p-value**
	**Conventional osteosynthesis**	**Jail technique**	
	**n = 10**	**n = 10**	
Maximum load	1670.69 ± 190.09	1858.06 ± 239.70	0.154
Displacement	3.63 ± 0.75	3.65 ± 0.85	0.073
Stiffness	437.52 ± 108.44	557.68 ± 76.08	0.785
Yield load	1465.16 ± 98.99	1516.95 ± 113.43	0.481

### Failure modes

**Figure 4:**
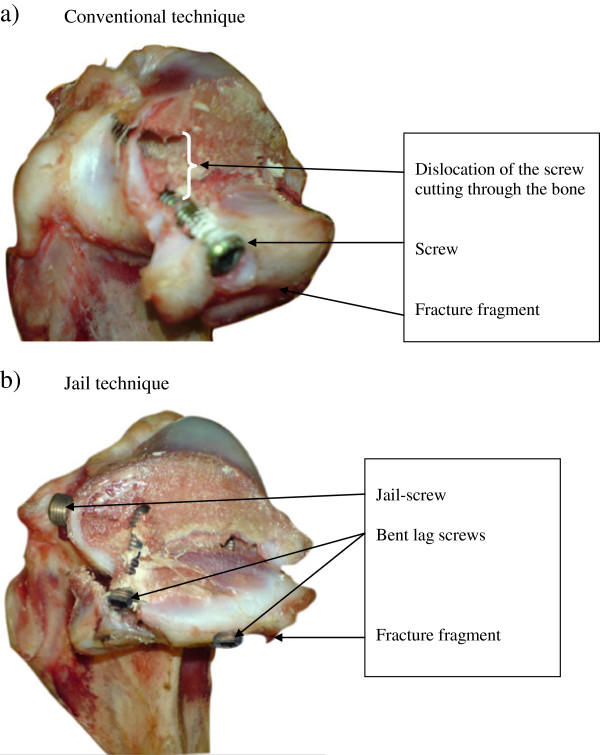
**The failure modes.** The figures show the failure mode in the two reconstructions; **a**) conventional reconstruction using a lateral two-screw osteosynthesis; **b**) jail technique using two lateral screws plus a jail screw. In Figure 4a, a screw was displaced as it cut through the bone. In Figure 4b, the lag screws were bent but no cut-out was observed.

The failure modes that occurred during the single cycle testing were similar to those observed during the cyclic load testing protocol. Although all reconstructions were loaded to failure, the typical failure modes differed between the two reconstruction groups as shown in Figure 4.

The predominant failure mode in the conventional osteosynthesis group was screws cutting through the bone (cut-outs) of the lateral tibial plateau (92%; Figure 4a). In the course of the experiment, the two lag screws were pushed downwards into the cancellous bone and bent slightly in the direction of force action. At the end of the test, a clear cutting through the tibial spongy bone was observed, an effect that was more evident in specimens tested cyclically than those tested in a single cycle.

In specimens reconstructed with the jail technique, a deformation of the two lateral screws at the site directly above the jail screw was documented as the typical failure mode (85%; Figure 4b) but no cut-outs in the cancellous bone were observed.

## Discussion

Conservative techniques of treating fractures of the tibial plateau were common in the past but many of these techniques resulted in poor outcomes and even caused lifelong disability in many cases [12]. A revolution in the operative treatments of these injuries was introduced by the AO Foundation [12,13], which led to the development of different reconstruction techniques [9,14], including minimally invasive options through arthroscopy. Because of these new developments, the number of reconstructions of tibial plateau fractures has increased significantly in recent years [9,13,14].

A minimally invasive technique using two parallel tension screws was first introduced by Schatzker et al. in 1979 [15]. Since then, minimally invasive arthroscopically-assisted procedures have become the norm in treating fractures like Schatzker Type I and AO Type B1 [16-19] similar to the fractures simulated and evaluated in this study.

This study evaluated 2 minimally invasive reconstruction techniques of lateral tibial plateau fractures, the conventional two-screw osteosynthesis vs. the novel three-screw jail technique.

The results of the present study could not confirm our hypothesis that fixation with a third additional screw as inserted in a jail technique could strengthen structural properties in the reconstruction of lateral tibial plateau fractures better than the conventional technique. However, there are some indications that the jail technique may be a feasible alternative to the conventional osteosynthesis fixation method.

In the single cycle loading tests, significant differences in maximal load were documented. Additionally the jail technique showed a trend for better outcomes in terms yield load, stiffness and displacement though the differences did not reach statistical significance.

In most of the variables measured during the two testing protocols, no statistically significant differences were observed between the two reconstruction techniques.

All reconstructions survived the cyclic loading tests regardless of fixation technique used. This lack of measurable differences suggests that the two reconstructions have similar biomechanical characteristics.

Despite the similarities between the two techniques, the different failure modes in the two reconstructions suggest that the jail technique may hinder the upper lag screws from cutting through the cancellous bone during loading. A possible explanation may be that the lag screws did not absorb the entire axial load force and transmit it to the cancellous bone. Instead, the additional abutment of the jail screw transmitted parts of the axial load into the cortical bone [20].

This study had several limitations. First, the bone mineral density of the porcine tibia is higher than that of the human tibia [10,21]. A high bone mineral density could theoretically lead to better biomechanical results. However, cadaver materials from donors who underwent tibial plateau reconstructions are hard to obtain and the low bone mineral density of older donors could lead to weaker biomechanical results.

Second, the experimental set up did not correlate to the physiological conditions in the clinical setting. The tibia was fixed statically and did not allow any movement. The load was applied axially in a worst-case scenario over the reconstructed tibial plateau. Structures such as the menisci were not considered. However, the experimental set up is a well-accepted procedure in orthopedic research [7,8,22,23].

Third, we investigated the material properties of the reconstructions under cyclic loading only at time of surgery. Fracture healing undergoes substantial remodeling during the postoperative period. Therefore, we only investigated the primary stability of the reconstruction techniques.

Fourth, we used a three-screw reconstruction for the jail technique and compared it to a two-screw osteosynthesis in the conventional technique. This was done because a conventional technique using three parallel screws is often not possible in matters of space on the lateral tibial plateau.

## Conclusions

The results of this study indicate that the jail and the conventional technique have seemingly similar biomechanical properties. This suggests that the jail technique may be a feasible alternative to conventional screw osteosynthesis in the minimally invasive reconstruction of lateral tibial plateau fractures. A potential ability of the jail technique is the prevention of screw cut-outs through the cancellous bone.

## Competing interests

The authors declare that they have no competing interests.

## Authors’ contributions

AW carried out the biomechanical studies and drafted the manuscript. TH carried out the biomechanical studies. MH carried out the statistical evaluation and corrected the manuscript. BD carried out the statistical evaluation and corrected the manuscript. WP participated in the study design and correction process. MJR participated in the study design and correction process. All authors read and approved the final manuscript.

## Pre-publication history

The pre-publication history for this paper can be accessed here:

http://www.biomedcentral.com/1471-2474/14/120/prepub
